# Determination of Chinese hamster ovary (CHO) cell densities and antibody titers from small volumes of cell culture supernatants using multivariate analysis and partial least squares regression of UV-Vis spectra

**DOI:** 10.1007/s00216-021-03549-4

**Published:** 2021-09-02

**Authors:** Salinthip Jarusintanakorn, Chutima Phechkrajang, Putthiporn Khongkaew, Enrico Mastrobattista, Montarop Yamabhai

**Affiliations:** 1 Utrecht Institute for Pharmaceutical Sciences (UIPS), Department of Pharmaceutics, Faculty of Science, Utrecht University, Universiteitsweg 99, 3584 CG Utrecht, Netherlands; 2 Department of Pharmaceutical Chemistry, Faculty of Pharmacy, Mahidol University, 447, Sri-Ayuthaya Road, Rajathevi, Bangkok, 10400 Thailand; 3 Molecular Biotechnology Laboratory, School of Biotechnology, Institute of Agricultural Technology, Suranaree University of Technology, Nakhon Ratchasima, 30000 Thailand; 4 Faculty of Pharmaceutical Science, Burapha University, 169 Longhaad Bangsaen Road, Saensook, Muang, Chonburi, 20131 Thailand

**Keywords:** Multivariate data analysis, Partial least squares regression, Viable cell density, Therapeutic antibody titer, CHO, UV-Vis spectroscopy

## Abstract

Antibody titer and viable cell density (VCD) are two important parameters that need to be closely monitored during the process of cell line development and manufacturing of therapeutic antibodies. Typically, determination of each parameter requires 10–100 μL of supernatant sample, which is not suitable for small scale cultivation. In this study, we demonstrated that as low as 2 μL of culture supernatants were sufficient for the analysis using UV-Vis spectrum assisted with partial least squares (PLS) model. The results indicated that the optimal PLS models could be used to predict antibody titer and VCD with the linear relationship between reference values and predicted values at *R*^2^ values ranging from 0.8 to > 0.9 in supernatant samples obtained from four different single clones and in polyclones that were cultured in various selection stringencies. Then, the percentage of cell viability and productivity were predicted from a set of samples of polyclones. The results indicated that while all predicted % cell viability were very similar to the actual value at RSEP value of 6.7 and *R*^2^ of 0.8908, the predicted productivity from 14 of 18 samples were closed to the reference measurements at RSEP value of 22.4 and *R*^2^ of 0.8522. These results indicated that UV-Vis combined with PLS has potential to be used for monitoring antibody titer, VCD, and % cell viability for both online and off-line therapeutic production process.

**Graphical abstract:**

**Figure:**
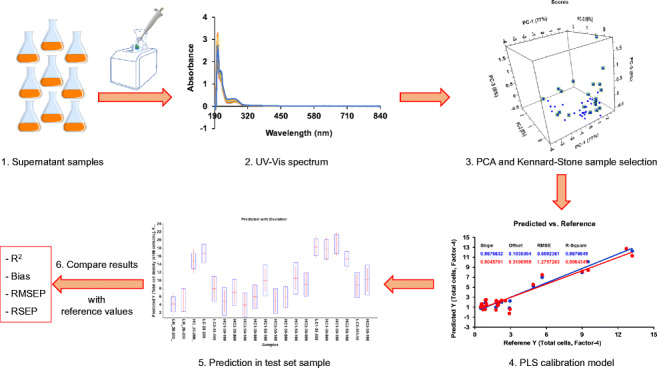
The process of multivariate analysis and partial least squares regression of UV-Vis spectrum for the determination of CHO cell densities and antibody titers obtained from small volume of cell culture supernatant samples.

## Introduction

Since the production of therapeutic antibodies involves the use of living cells, the manufacturing process is much more complicated than generating small molecule drugs, which requires chemical reactions [1]. Multiple bioprocess parameters such as viable cell density (VCD), antibody titer, pH, temperature, %CO_2_, and level of metabolites need to be tightly monitored in order to obtain maximum yield and to ensure consistency of product quality [2]. These bioprocess parameters are necessary to monitor the performance of cell lines both during the process development and routine manufacturing [3]. Among these parameters, VCD and antibody titers are the two key parameters for determining optimal condition for cell line cultivation during bioprocess development [4]. However, monitoring of these parameters need specific methods and instruments, which are expensive, laborious [5], require substantial volume of samples [6], or sophisticated instruments in case of real-time measurement [7, 8]. For example, the determination of antibody titers by ELISA [9] involves several reagents and a microplate reader, and the determination of %viability by trypan blue staining requires a cell counter or inverted microscope as well as experienced personnel [10].

Recently, multivariate data analysis (MVDA) such as principal component analysis (PCA) and partial least squares (PLS), in conjunction with spectroscopic spectra, has been considered to be a powerful tool in the process analytical technology (PAT) or quality by design (QbD) of biopharmaceutical processes [11, 12]. The most popular spectroscopies combined with MVDA in PAT tool are Raman spectroscopy [13, 14], near-infrared spectroscopy (NIR) [15], and Fourier-transform infrared spectroscopy (FTIR) [16]. This is because these spectroscopic methods can be used to identify chemical functional groups [17]. Ultraviolet-Visible (UV-Vis) spectra provide less information when compared with other spectroscopy techniques as the bands are less specific and have high chances of spectral overlapping [17]. However, this method is more attractive in terms of ease of handling and cost, which is more suitable for lab-scale or manufacturing of therapeutic antibody in low-to-middle-income countries. There has been a previous report on successful application of this technique to monitor glutamine and glucose concentrations in BHK-21 cell cultures [18].

In this study, the potential of monitoring VCD and antibody titers from lab-scale Chinese hamster ovary (CHO) cell cultures by applying UV-Vis spectra in combination with MVDA and PLS calibration model was demonstrated. The data was obtained from an off-line UV-Vis spectrum of samples from lab-scale CHO cell culture supernatants.

## Materials and methods

### Reagents and materials

Dynamis (catalog no. A2661501), CD FortiCHO (catalog no. A1148301), and anti-clumping agent (catalog no. 0010057AE) were purchased from Thermo Fisher Scientific (Waltham, MA, USA). For ELISA method, Invitrogen™ Nunc MaxiSorp™ flat-bottom 96-well plates (catalog no. 44-2404-21) were purchased from Thermo Fisher Scientific (Waltham, MA, USA). Protein A (catalog no. GSZ02201) was purchased from GenScript (Piscataway, NJ, USA). Peroxidase AffiniPure F(ab′)_2_ fragment goat anti-human IgG (H + L) HRP (catalog no. 109-036-088) was purchased from Jackson Immuno Research Laboratories (West Grove, PA, USA). ABTS (Diammonium 2,2′-azinobis[3-ethyl-2,3-dihydrobenzothiazole-6-sulfonate]) was ordered from VWR (Tuas, Singapore). TMB (3,3′,5,5′-tetramethylbenzidine) solution (catalog no. 34028) was purchased from Thermo Fisher Scientific (Waltham, MA, USA).

### Cell culture

Therapeutic antibody-producing CHO cells were developed and cultured using the platform of the Molecular Biotechnology Laboratory (MY Lab) at Suranaree University of Technology (Thailand), which is based on CHO-S system, using DHFR and puromycin as selection markers (Thermo Fisher Scientific, Waltham, MA, USA, catalog # A1155701). Four different single clones were cultured separately in 125-mL shaker flasks with Dynamis™, supplemented with 8 mM of l-glutamine and anti-clumping agent (1:1000 dilution). Polyclones were cultured in CD FortiCHO™, supplemented with 8 mM of l-glutamine, anti-clumping agent (1:100 dilution), various concentrations of methotrexate (MTX) (100, 200, 500, and 1000 nM), and puromycin (10, 20, 30, and 50 μg/mL). Some supernatant samples also contained 4–6 g/L of glucose supplements, according to the MY Lab selection protocol, which was modified from productivity assessment guideline of Freedom™ CHO-S kit (Thermo Fisher Scientific, Waltham, MA, USA). Supernatant samples obtained from these cell cultures were kept at 4 °C until further analysis.

### Cell counting

The viable and dead cell densities were counted on a Neubauer chamber by mixing trypan blue dye solution with proper dilution of culture sample. Dead cells appeared blue.

### Determination of antibody titer and productivity

Determination of antibody titer in supernatant samples was achieved by ELISA in Nunc MaxiSorp™ flat-bottom 96-well plate. Firstly, wells of the 96-well plate were coated with Protein A solution at 4 °C overnight. Then, the excess amount of Protein A was removed by three-time washings with washing buffer (0.05% Tween 20 in PBS) before incubating the wells with blocking buffer (1% BSA in washing buffer) at room temperature (RT) for 1 h. After three-time washings to remove excess blocking buffer, supernatant samples and different concentrations of Humira® were added to each well and incubated for 1 h, at RT. Following three washings of the plate, Peroxidase AffiniPure F(ab′)_2_ fragment goat anti-human IgG (H + L) HRP (a labeled detection antibody) was added to each well and incubate at RT for 1 h. After that, the plate was washed three times again before the substrate was added into each well. At this step, two different substrates, i.e., ABTS solution (1 mM ABTS in 50 mM Citric acid containing 0.01% H_2_O_2_) and TMB solution, were used for the detection of antibody from supernatant samples obtained from four different single clones and polyclones, respectively. For detection using ABTS, the plate was incubated at RT in the dark for 30 min before adding 1% SDS solution to stop the reaction; while for TMB, the reaction was incubated at RT in the dark for 20 min before 2 M H_2_SO_4_ was added to stop the reaction. After the reaction was stopped, the microplate reader (Sunrise™, Tecan, Männedorf, Switzerland) was set to measure the absorbance at 405 and 450 nm for ABTS and TMB, respectively.

Productivity (pg/cell/day) was calculated based on antibody titer and integral viable cell density (IVCD) [19] as shown in the following equations.

1 $$ {\mathrm{IVCD}}_t=\frac{1}{2}\times \left({\mathrm{VCD}}_t+{\mathrm{VCD}}_{t_0}\right)\times \Delta  t+{\mathrm{IVCD}}_{t_0} $$

2 $$ \mathrm{Productivity}\ \left(\mathrm{pg}/\mathrm{cell}/\mathrm{day}\right)=\frac{\mathrm{Antibody}\ \mathrm{titer}}{\mathrm{IVCD}} $$

where IVCD is integral viable cell density, VCD is viable cell density, *t* is cultured time, *t*_0_ is initial cultured time, and Δ*t* is time interval.

### UV-Vis measurement

Supernatant samples were diluted 5-fold with deionized water. Then, the UV-Vis spectra of these diluted samples were measured at the wavelengths of 190–840 nm by NanoDrop™ 2000 Spectrophotometer (Thermo Fisher Scientific, Waltham, MA, USA), using deionized water as a blank. The absorbance data were collected every 1 nm and exported to excel file for further analysis.

### Data pre-treatment and multivariate modeling

Unscrambler X version 10.4 (Camo Analytics, Oslo, Norway) was used to analyze the absorbance data of 4 different single clones for multivariate modeling. Before analysis, data pre-treatment was performed by subtracting the absorbance data with the cultivating medium. The first step of multivariate modeling was to employ principal component analysis (PCA) to reduce the dimensionality of the dataset [20, 21]. Then, the Kennard-Stone algorithm [22] was used to select samples for the calibration set, and the remaining samples were used as a test set. After sample classification, each PLS model was constructed for each variable. To ensure that results generated by the model are relevant and correct, the model is internally cross-validated by random cross-validation method (with leave two out and twenty segments) and externally validated with the independent test set samples (validation samples); those were not used to develop the model. The reliability of the model was evaluated based on the *R*^2^ of the model from calibration step, *R*^2^ Pearson from validation step, root mean square error of prediction (RMSEP), relative standard error of prediction (RSEP), and bias values. For the calculation for RMSEP, RSEP, and bias, the following equations were used:

3 $$ \mathrm{RMSEP}=\sqrt{\frac{\sum_{\mathrm{i}=1}^n{\left({\mathrm{y}}_{\mathrm{pred}}-{\mathrm{y}}_{\mathrm{ref}}\right)}^2}{\mathrm{n}}} $$

4 $$ \mathrm{RSEP}=100\ \sqrt{\frac{\sum_{\mathrm{i}=1}^n{\left({\mathrm{y}}_{\mathrm{pred}}-{\mathrm{y}}_{\mathrm{ref}}\right)}^2}{\sum_{\mathrm{i}=1}^n{\left({\mathrm{y}}_{\mathrm{ref}}\right)}^2}} $$

5 $$ \mathrm{Bias}=\kern0.5em \mathrm{Average}\ \left({\mathrm{y}}_{\mathrm{pred}}-{\mathrm{y}}_{\mathrm{ref}}\right) $$

where y_pred_ is predicted value, y_ref_ is reference value, and *n* is number of samples.

## Results and discussion

**Fig. 1:**
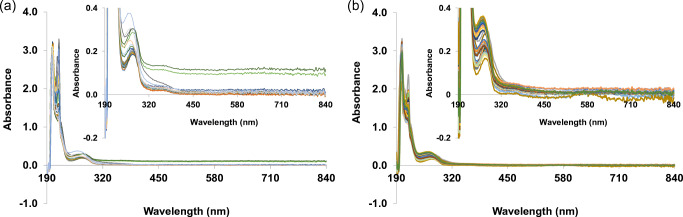
UV-Vis spectrum of 46 (**a**) and 70 (**b**) supernatant samples obtained from 4 different single clones and 14 polyclones cultures, respectively. Insets are the enlargement of peaks with low absorbance. Different supernatant samples are represented as different line colors

A univariate or bivariate analysis from UV-Vis spectrum of typical supernatant samples indicated that these methods could not be used to quantify the amount of antibody or monitor cell growth in culture supernatant of either single or polyclones, as illustrated in Fig. 1. This difficulty was probably due to the presence of various compounds such as nutrients from cultured medium, supplements, metabolites, produced antibody, and compounds that are used for the selection of transfected clones. Therefore, in this study, MVDA was used to predict antibody titer and cell viability profile. The investigations were conducted on four different single clones that were cultured in the same culture conditions, and 14 polyclones that were cultured under 4 different MTX selection pressures; a total of 70 samples were used in the assay.

### Analysis of supernatant samples obtained from 4 different individual clones

**Fig. 2:**
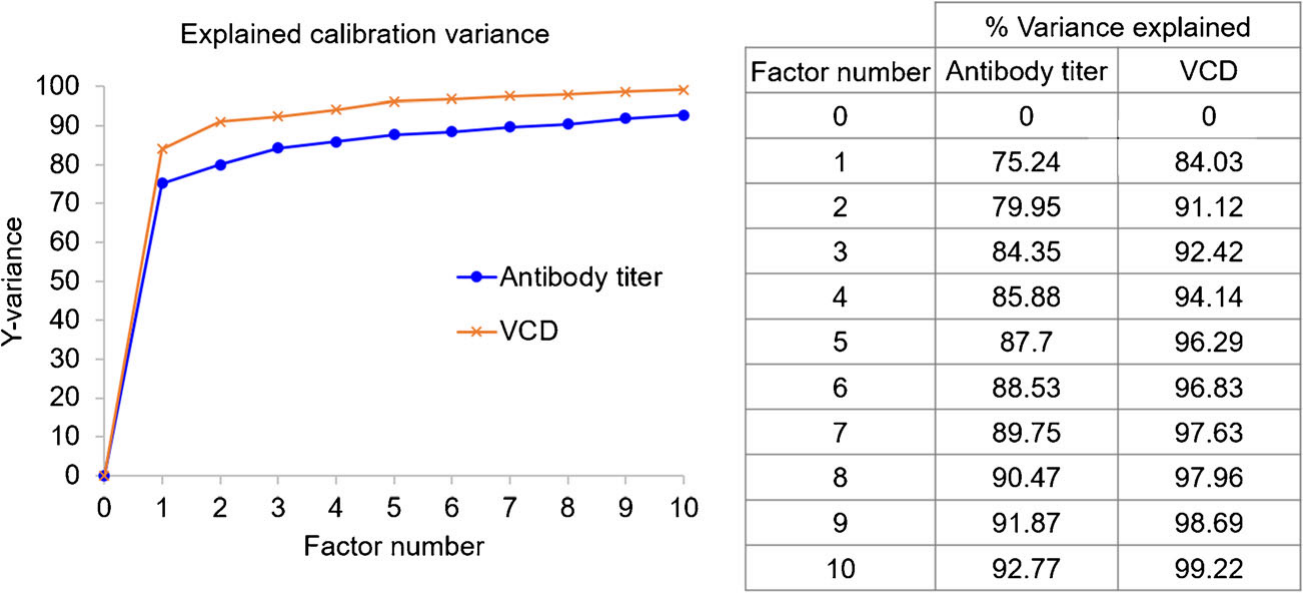
The percentage of variance explained as a function of number of factors used in PLS models for the prediction of antibody titer and VCD of cultures of four different single clones

**Fig. 3:**
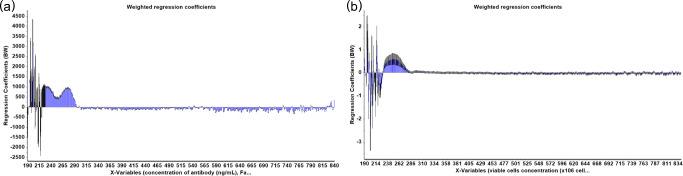
The weight regression coefficients of PLS models for the prediction of antibody titer (**a**) and VCD (**b**) of cultures of four different single clones

**Fig. 4:**
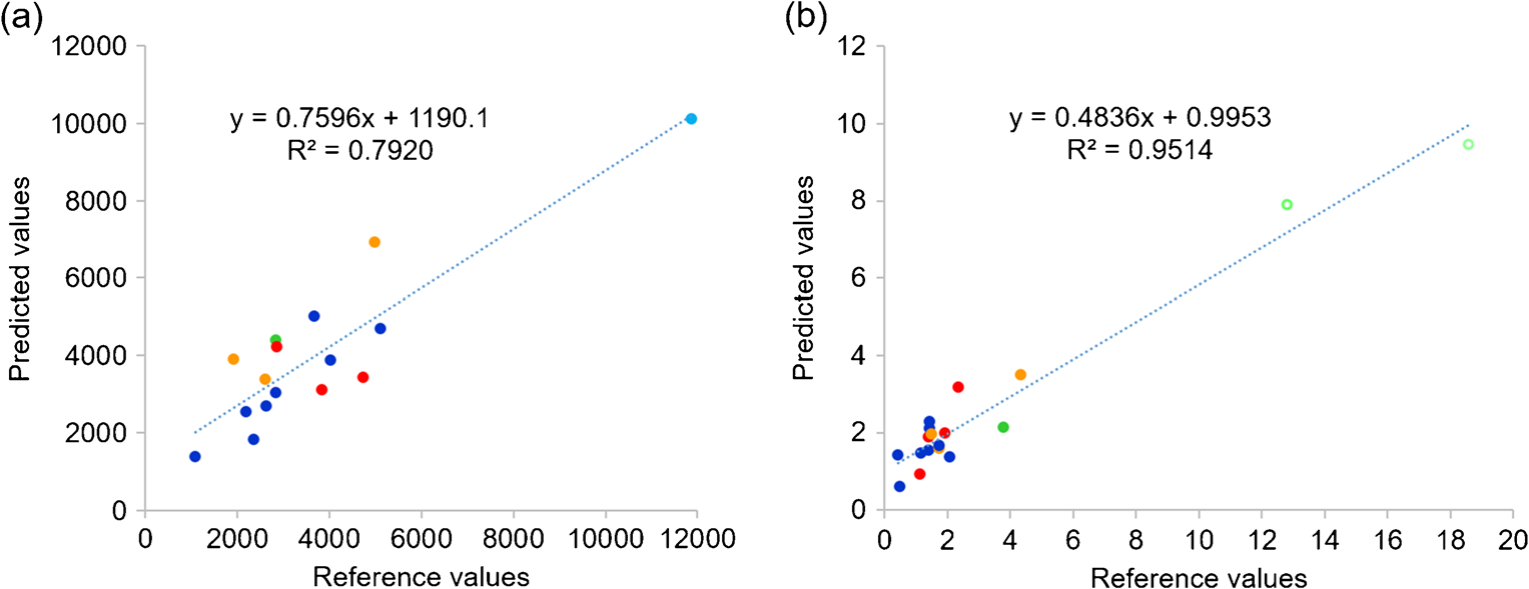
Predicted antibody titer (**a**) and viable cell density (**b**) from the test set samples. Red, green blue, and orange dots represent the 4-single clones; while the light green open circles represent single clones that were cultured in media with glucose supplements

A summary of statistical parameters for PLS modeling of isolated single clones are shown in Table 1. From 48 samples obtained from different passages of four different single clones, 30 samples were used to construct the calibration model for the prediction of antibody titer and only 27 samples were used to construct the calibration model for the prediction of VCD because 3 samples were outlier sample and was not selected for the analysis. PLS is a latent projection approach [23], which transform a large number of correlated variables (here mean the UV wavelengths), into a possibly smaller number of uncorrelated variables (latent variables or latent factors). The optimal calibration model should be obtained from the minimum latent factor with suitable *R*^2^ Pearson and model error parameters in order to avoid over-fitting of the calibration model. The correlation between reference and predicted values in term of *R*^2^ of the model was used to estimate model performance. The *R*^2^ closed to 1 was the estimation criteria. In this study, five and seven latent factors were used for the prediction of VCD and antibody titer to achieve *R*^2^ value greater than 0.95 and 0.89, respectively (Table 1). The values of %variance explained and weight regression coefficient of these two PLS models are displayed in Figs. 2 and 3, respectively. Then, the PLS model for each variable was applied to predict the variable in the test set samples. The results showed a good correlation (linear relationship) between the predicted values obtained from PLS model and the actual measured values obtained from the reference method, with *R*^2^ = 0.9514 and 0.7920 for the predicted VCD and antibody titer, respectively (Fig. 4, Table 2). These results indicated that UV-Vis spectrum assisted with MVDA could be used to predict VCD value better than antibody titer, as indicated by the *R*^2^ value. These results might be due to the fact that the structure of the antibody is quite complex and supernatant samples might contain a small degree of aggregates that could interfere with the UV-Vis spectra [24]. Furthermore, the ELISA method that was used as the reference method could only detect properly folded antibodies, and therefore, an HPLC method might probably provide a more reliable estimate for the concentration of antibody [25, 26]. In addition, to estimate model performance, RMSEP, RSEP, and bias were used to measure accuracy and precision of the model. The lowest values of these performance characteristics indicate the reliability of the model. Units of RMSEP and bias were the same as the predicted variable; whereas, RSEP was expressed as percentage of standard error of prediction. From the results reported in Table 2, the calibration model for the prediction of VCD showed higher error of prediction than antibody titer, as indicated by high RSEP value. This higher error might be because of other compounds such as glucose supplement and metabolites in the supernatant samples of fed-batch culture that might affect the prediction of VCD. Notably, the prediction of samples obtained from cell culture with glucose supplement was not as good as those without glucose (light green open circle in Fig. 4b). This might be because low number of samples with glucose supplement was available for building the calibration model. More numbers of single clones or samples with glucose supplement containing different antibody concentrations in the calibration set may help improving the predictability of the calibration model especially for VCD.

**Table 1:** PLS model statistics for antibody titer and VCD from 30 samples obtained from 4 different single clone cultures

Parameters	Variables	
	Antibody titer (ng/mL)	VCD (× 10^6^ cells/mL)
Number of samples in calibration set	30	27*
Latent factors	7	5
Linear model parameter		
- Slope	0.8975	0.9629
- Offset	365.85	0.0953
- Correlation coefficient (*R*^2^)	0.8975	0.9629

*Three samples were defined as outlier samples and remove from analysis

**Table 2:** Summary of statistical parameters of the test set of 4 different single clones

Statistical parameters	Variables	
	Antibody titer (ng/mL)	VCD (× 10^6^ cells/mL)
Number of samples in test set	16	18
Slope	0.7596	0.4836
Offset	1190.1	0.9953
*R*^2^ Pearson	0.7920	0.9514
Bias	293.8	− 0.7
RMSEP	1125.6	2.5
RSEP	25.5	44.6

### Analysis of supernatant samples of polyclones from various culture conditions

**Fig. 5:**
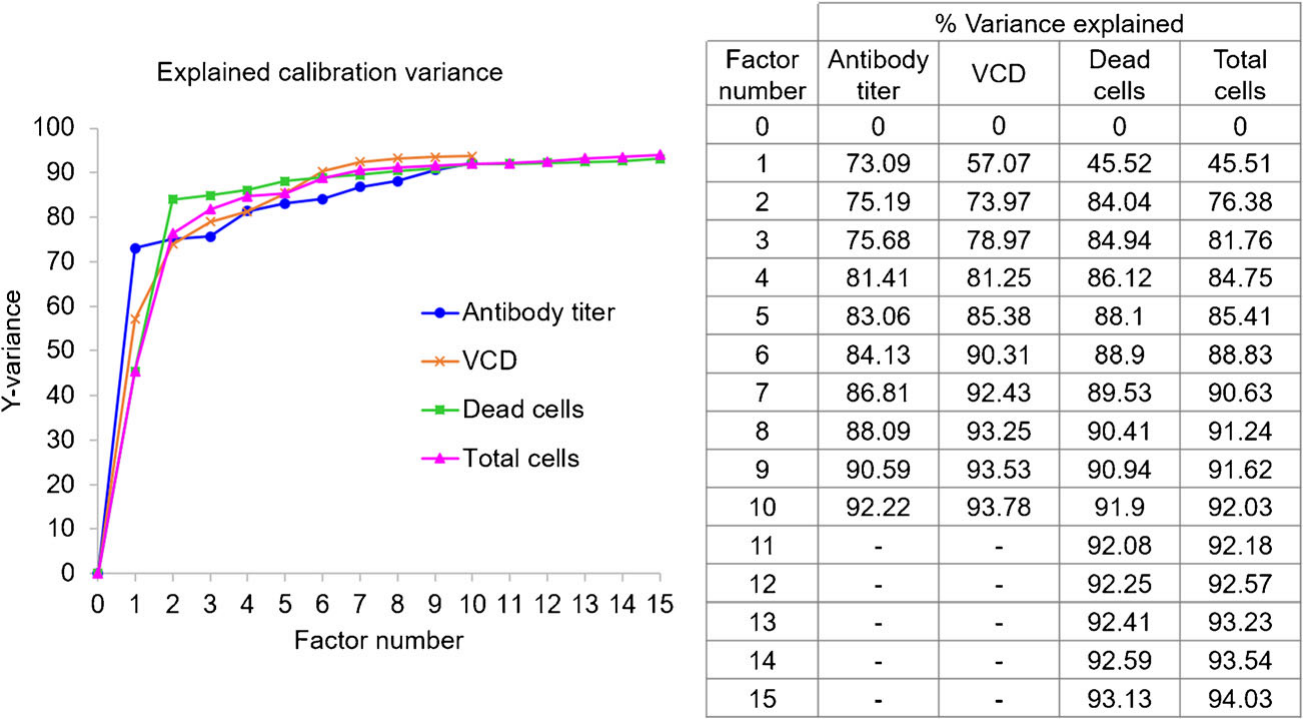
The percentage of variance explained as a function of number of factors used in PLS models for the prediction of antibody titer, VCD, dead cells, and total cells of polyclones cultures

**Fig. 6:**
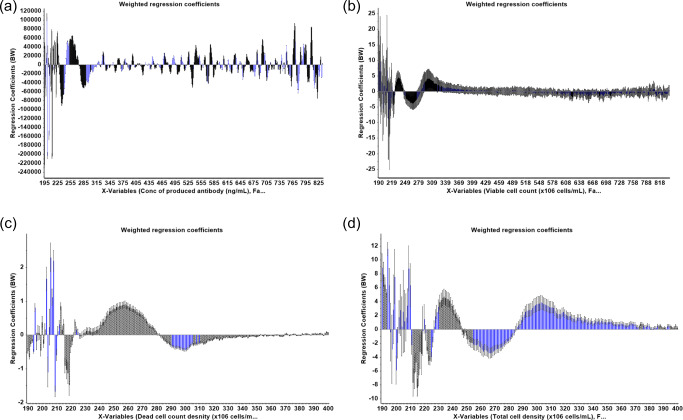
The weight regression coefficients of PLS models for the prediction of antibody titer (**a**), VCD (**b**), dead cells (**c**), and total cells (**d**) of polyclone cultures

**Fig. 7:**
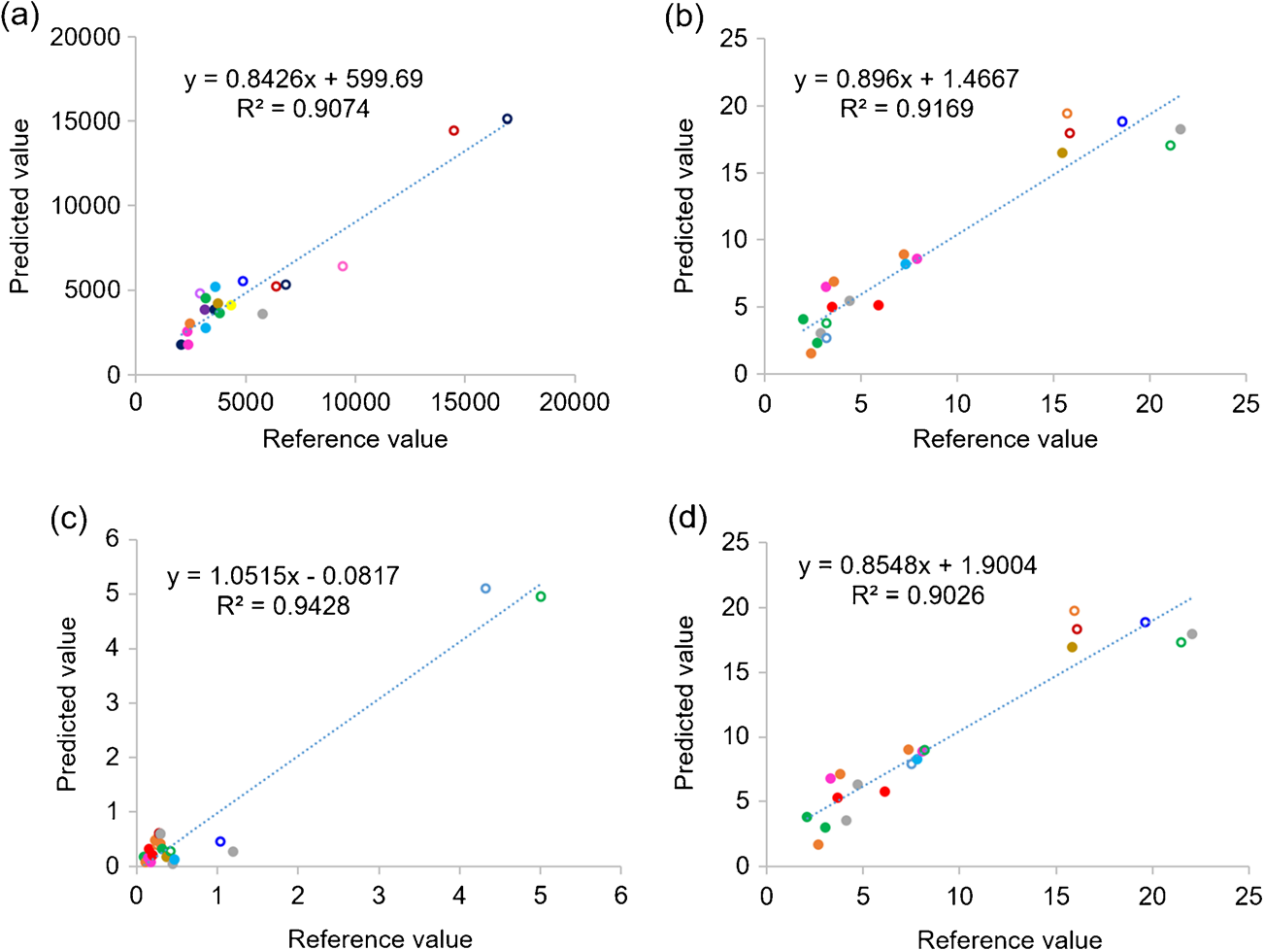
Predicted antibody titer (**a**), viable cell density (VCD) (**b**), dead cell density (**c**), and total cell density (**d**) from the test set samples (each dot color represents each pool of polyclones and the open circles represent the culture condition with glucose supplements)

A critical step to efficiently produce therapeutic antibody is cell line development. During this step, different stringent selection pressures are applied to pools of transfected cells or polyclones in order to enhance antibody titer before the isolation of single clones. Monitoring percentage cell viability, VCD, and antibody titers are crucial for performing an effective selection procedure. In this experimental section, 70 supernatant samples obtained from 14 different pools of polyclones that were treated differently during the process of stable cell line selection were used for analysis. Fifty samples (from 14 different pools of polyclones) were selected by Kennard-Stone sample selection method to build a model for the prediction of four different parameters, namely, antibody titer, VCD, dead cell density, and total cell density. The remaining 20 samples, which came from 8 different pools of polyclones, were used as a test set sample. For antibody titer prediction, 5 samples were defined as outlier samples and removed from the analysis. Therefore, the PLS calibration model was constructed from 45 samples, using 9 latent factors to achieve *R*^2^ value greater than 0.9 (Table 3). In case of cell viability profile, consisting of VCD, dead cell, and total cell density, 50 samples were used to construct the PLS calibration model for each variable with *R*^2^ > 0.9 (Table 3). The values of % variance explained and weight regression coefficient of PLS models are displayed in Figs. 5 and 6, respectively. A linear relationship between the predicted antibody titer obtain from the PLS calibration model and actual antibody titer could be achieved as shown in Table 4 and Fig. 7a. For cell viability profile, the prediction results suggested that each PLS model could be used to predict the assigned parameters with good linear correlation (*R*^2^ > 0.9) (Table 4 and Fig. 7b–d). Additionally, all models of polyclones showed good performance with acceptable RSEP values, ranging from 18.8 to 22.8. Normally, RSEP < 20% is preferable [27, 28]; however, RSEP ≤ 25% was acceptable in some reports [29, 30]. These results supported practical applications of the developed PLS models for new coming samples. Therefore, it could be concluded that PLS, which is one of the most popular MVDA technique [31], can help to deconvolute UV-Vis spectra for the determination of antibody titer and cell density profile, including VCD, dead cell, and total cell density in the supernatant samples of polyclones that were cultured at different levels of selection pressure during the selection process of cell line development for therapeutic antibody manufacturing.

**Table 3:** PLS model statistics for antibody titer, VCD, dead cell density and total cell density from 50 samples obtained from polyclones cultures

Parameters	Variables			
	Antibody titer (ng/mL)	VCD (× 10^6^ cells/mL)	Dead cell density (× 10^6^ cells/mL)	Total cell density (× 10^6^ cells/mL)
Number of samples in calibration set	45*	50	50	50
Latent factors	9	7	6	7
Linear model parameter				
- Slope	0.9059	0.9243	0.8890	0.9063
- Offset	539.02	0.6463	0.2079	0.9725
- Correlation coefficient (*R*^2^)	0.9059	0.9243	0.8890	0.9063

*Five samples were defined as outlier samples and removed from the analysis

**Table 4:** Summary of statistical parameters from the test set of polyclones

Statistical parameters	Variables			
	Antibody titer (ng/mL)	VCD (× 10^6^ cells/mL)	Dead cell density (× 10^6^ cells/mL)	Total cell density (× 10^6^ cells/mL)
Number of samples in test set	20	20	20	20
Slope	0.8426	0.8960	1.0515	0.8548
Offset	599.69	1.4667	− 0.0817	1.9004
*R*^2^ Pearson	0.9074	0.9169	0.9428	0.9026
Bias	− 232.45	0.5935	− 0.04	0.5662
RMSEP	1242.5	2.0	0.4	2.1
RSEP	18.9	18.8	22.8	19.0

### Determination of the percentage of cell viability and productivity using predicted values obtained from supernatant samples of polyclones

**Fig. 8:**
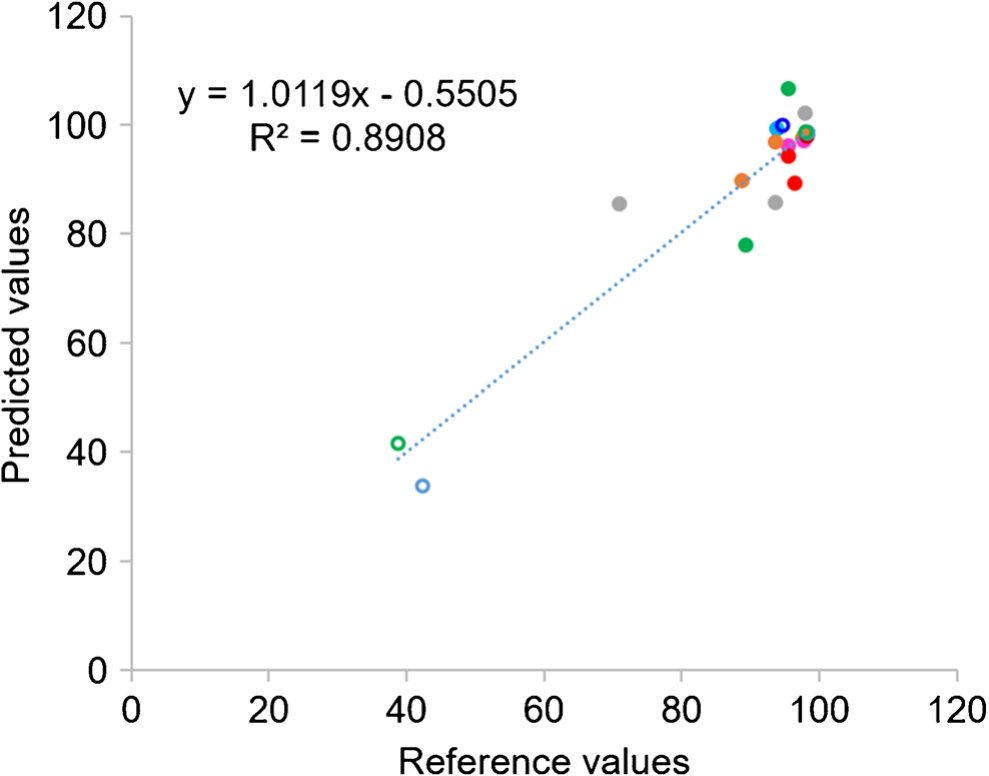
Predicted and reference values of % cell viability from 20 samples (each dot color represents each pool of polyclones and the open circles represent the culture condition with glucose supplements)

**Fig. 9:**
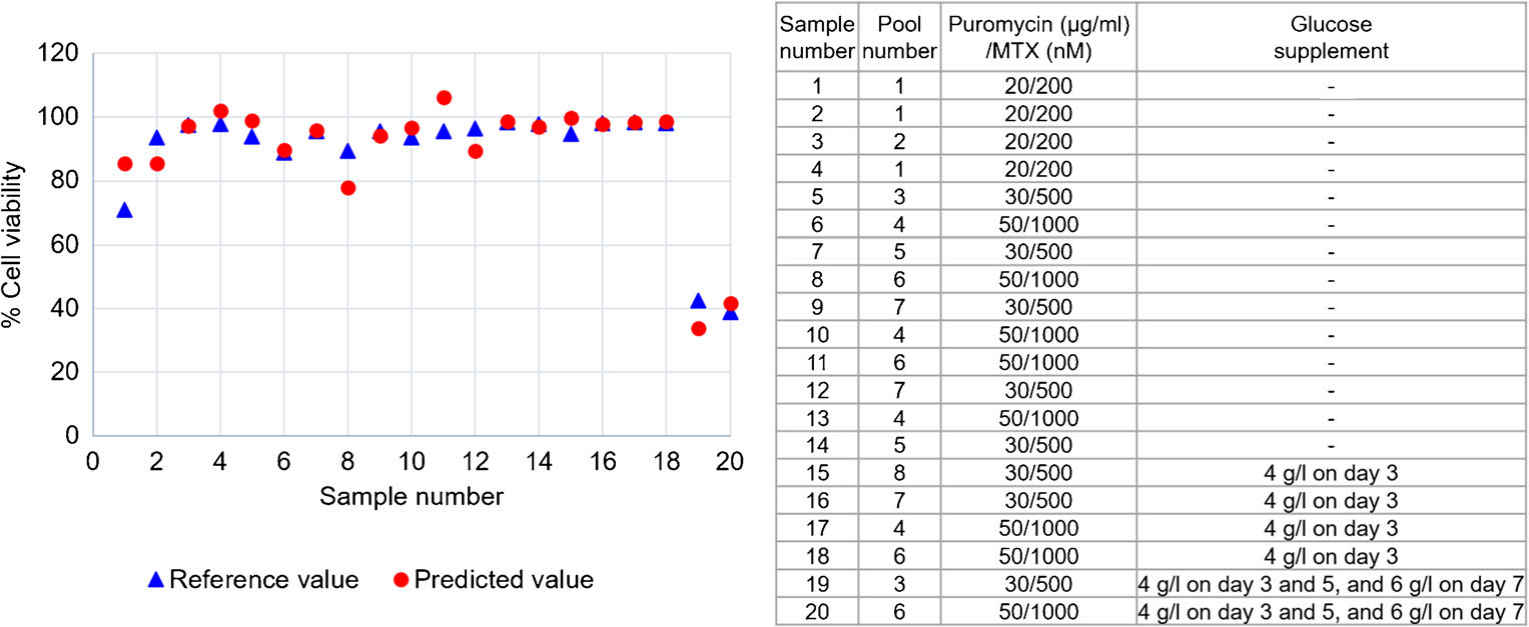
Predicted % cell viability of 20 samples, obtained from 8 different pools, in various culture conditions. Details of culture conditions of each sample are showed in the inset table

Finally, the predicted VCD and total cell density values from the test set samples of polyclones were further used to calculate the percentage of cell viability and productivity, which were then compared with the reference values determined from actual cell counting by trypan blue staining. As shown in Figs. 8 and 9, the predicted values of % cell viability were closed to the reference values with *R*^2^ at 0.8908 and RSEP of 6.7 (Table 5). Comparison of predicted and actual values of 20 test samples, from 8 different pools of polyclones, which were cultured in different selection stringencies (indicated in the inset table) are shown in Fig. 9. These results suggested that UV-Vis measurement of supernatant combined with PLS models of VCD and total cell density can be used to predict % cell viability. By employing this method, it is possible to use only 2 μL of supernatant samples to determine % cell viability as opposed to the trypan blue technique, which require 5–10 μL of the samples and is more time-consuming. Using low volume of supernatant samples to count number of cells will be very helpful for the calculation of productivity (pg/cell/day) in the small scale such as in a 96-well plate that is normally used during the limiting dilution step to select for single stably expressed clone.

**Table 5:** Summary of statistical parameters for the prediction of percentage of cell viability

Statistical parameters	% Cell viability
Number of samples	20
Slope	1.0119
Offset	− 0.5505
*R*^2^ Pearson	0.8908
Bias	0.5
RMSEP	6.1
RSEP	6.7

**Fig. 10:**
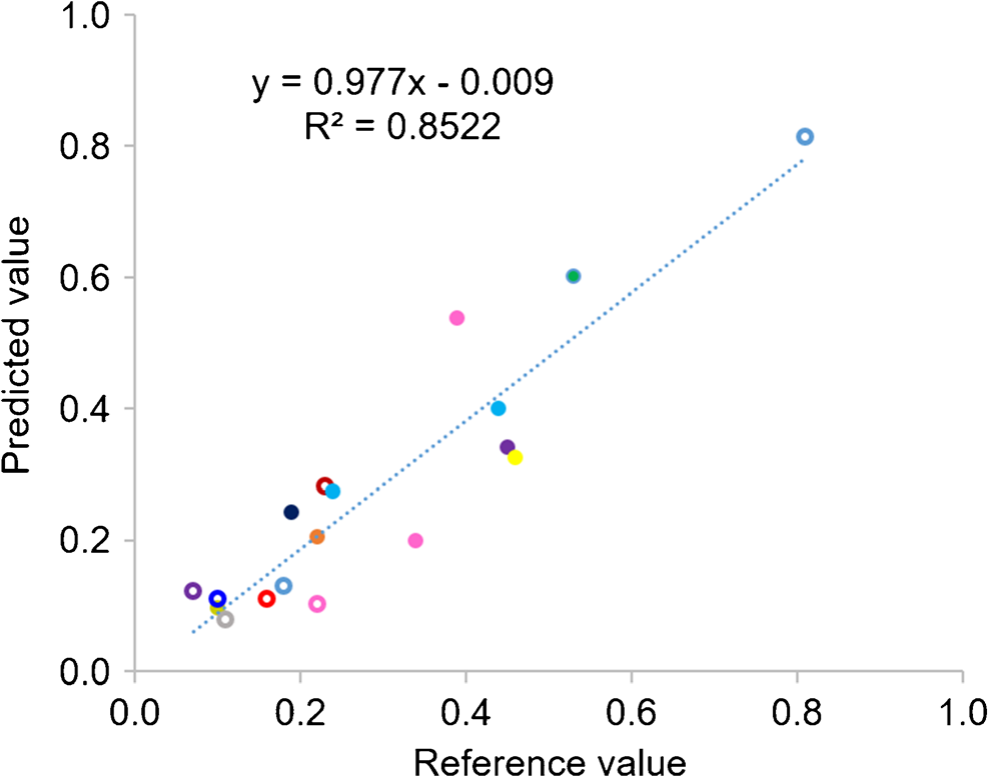
Predicted and reference values of antibody productivity from 18 samples. Each dot color represents each pool of polyclones, and the open circles represent the culture condition with glucose supplements

**Fig. 11:**
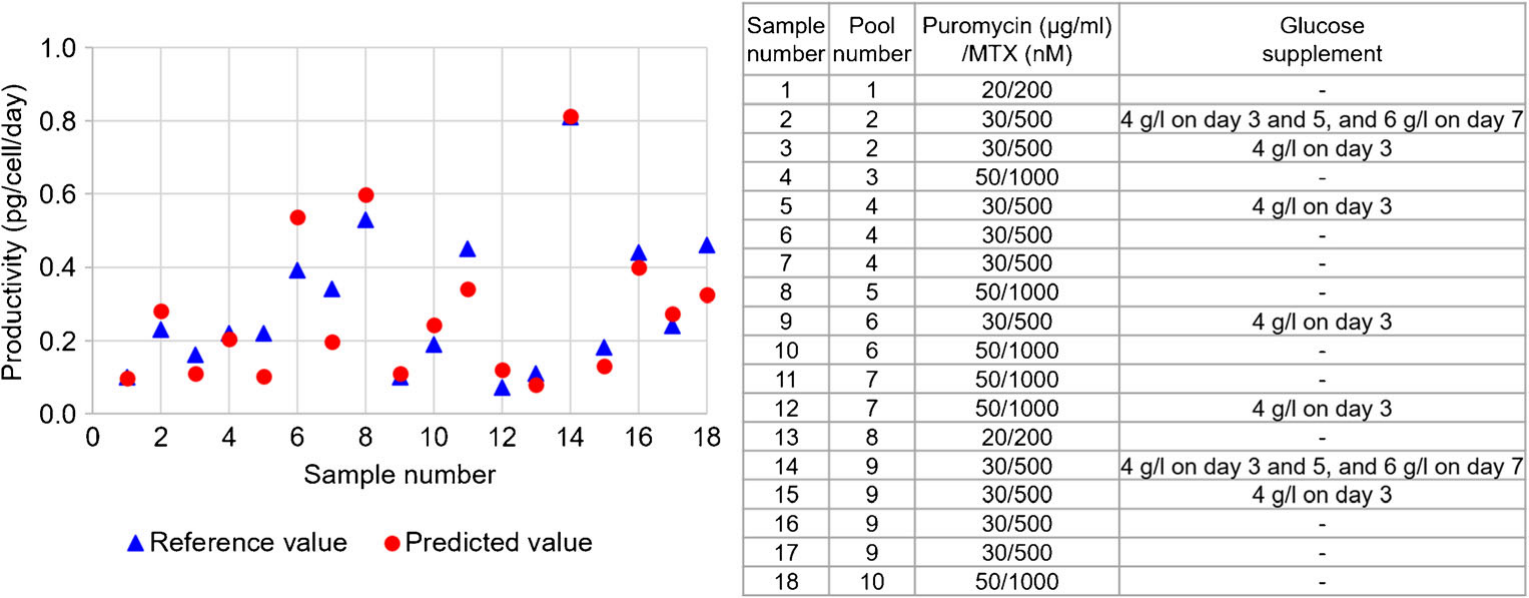
Predicted antibody productivity of 18 samples, obtained from 10 different pools, in various culture conditions. Details of culture conditions of each sample are showed in the inset table

For the prediction of antibody productivity, 18 samples from the test set samples, obtained from 10 different pools of polyclones, were used. The predicted productivity was calculated based on the predicted values of VCD and antibody titer and compared with the reference values. The results showed low bias at − 0.02 as illustrated in Fig. 10, with *R*^2^ at 0.8522 (Table 6). Comparison of predicted and actual productivities of 18 test samples, from 10 different pools of polyclones, which were cultured in different selection stringencies (indicated in the inset table), are shown in Fig. 11. The results indicated that 14 of 18 samples showed predicted values of productivity closed to those of the reference values at RSEP of 22.4 (Table 6), whereas the prediction results of the remaining 4 samples were not satisfactory. These might be due to the large deviation of predicted VCD values from the reference values for sample numbers 6, 7, and 18, and significant deviation of predicted antibody titer for sample number 5. Therefore, more sample and optimization must be done to successfully use this method to predict antibody productivity in CHO cell cultures.

**Table 6:** Summary of statistical parameters for the prediction of antibody productivity

Statistical parameters	Productivity
Number of samples	18
Slope	0.9770
Offset	− 0.009
*R*^2^ Pearson	0.8522
Bias	− 0.02
RMSEP	0.08
RSEP	22.4

Lastly, another attractive application of this technique is for off-line monitoring of cell growth instead of using the online viable biomass probe that is costly and required approximately 20–30 ml of the cell culture to calibrate the probe before it can be used to monitor cell growth in the bioreactor, of which further optimization of a suitable model must be performed.

## Conclusion

Antibody titer, VCD, and % cell viability are crucial monitoring parameters of the therapeutic antibody production process. Based on this work, a combination of UV-Vis and MVDA could be used to predict antibody titers and viable cell density in supernatant samples and this technique could also be applicable for the prediction of % cell viability and productivity in supernatant samples. It is worthwhile to highlight that the key advantage of this technique is the amount of volume required which is as low as 2 μL. Moreover, the costs related to ELISA technique for the quantification of antibody titer could be omitted. Therefore, this study presents the possibility of using off-line UV-Vis in combination with MVDA at the lab scale for routine monitoring of therapeutic antibody production at various manufacturing steps at lower cost and less time-consuming.

## Data Availability

Available upon request.
